# Psychosocial support during childbirth: Development and adaptation of WHO’s Mental Health Gap Action Programme (mhGAP) for maternity care settings

**DOI:** 10.1371/journal.pone.0285209

**Published:** 2023-05-22

**Authors:** Bushra Khan, Waqas Hameed, Bilal Iqbal Avan

**Affiliations:** 1 Department of Psychology, University of Karachi, Karachi, Pakistan; 2 Department of Community Health Sciences, Aga Khan University, Karachi, Pakistan; 3 Department of Population Health, Faculty of Epidemiology and Population Health, London School of Hygiene and Tropical Medicine, London, United Kingdom; Kasturba Medical College Mangalore, Manipal Academy of Higher Education, INDIA

## Abstract

**Introduction:**

Poor psychosocial support and lack of respectful care for women during childbirth are commonplace in health facilities in low- and middle-income countries. While WHO recommends providing supportive care to pregnant women, there is a scarcity of material for building the capacity of maternity staff to provide systematic and inclusive psychosocial support to women in the intrapartum phase, and prevent work stress and burnout in maternity teams. To address this need we adapted WHO’s mhGAP for maternity staff to provide psychosocial support in labour room settings in Pakistan. Mental Health Gap Action Programme (mhGAP) is an evidence-based guidance which provides psychosocial support in resource-limited health care settings. This paper aims to describe the adaptation of mhGAP to develop psychosocial support capacity building materials for maternity staff to provide support to maternity patients, and also to staff, in the labour room context.

**Methods:**

Adaptation was conducted within the Human-Centered-Design framework in three phases: inspiration, ideation, and implementation feasibility. In inspiration, a review of national-level maternity service-delivery documents and in-depth interviews of maternity staff were conducted. Ideation involved a multidisciplinary team to develop capacity-building materials by adapting mhGAP. This phase was iterative and included cycles of pretesting, deliberations, and revision of materials. In implementation feasibility, materials were tested via the training of 98 maternity staff and exploring system feasibility via post-training visits to health facilities.

**Results:**

Inspiration phase identified gaps in policy directives and implementation and formative study identified limited understanding and skills of staff to assess patients’ psychosocial needs and provide appropriate support. Also, it became evident that staff themselves needed psychosocial support. In ideation, team developed capacity-building materials comprising two modules: one dedicated to conceptual understanding, the other to implementing psychosocial support in collaboration with maternity staff. In implementation feasibility, staff found the materials relevant and feasible for the labour room setting. Finally, users and experts endorsed usefulness of the materials.

**Conclusion:**

Our work in developing psychosocial-support training materials for maternity staff extends the utility of mhGAP to maternity care settings. These materials can be used for capacity-building of maternity staff and their effectiveness can be assessed in diverse maternity care settings.

## Introduction

Disrespect and lack of psychosocial support in health facilities during childbirth are common phenomena in low- and middle-income countries [1]. Childbirth is a unique experience for women, and their memories of it are indelible [2]. There is an evidence that women consider childbirth traumatic [3]. Those who experience distress and non-supportive care, on top of the inevitable stress, during childbirth are left at risk of developing postnatal mental health issues [4]. Moreover, women with pre-existing mental health issues—particularly depression or anxiety—or physical disability, or socio-demographic vulnerabilities in relation to education, age and socio-economic status are disproportionately at risk for poor care [5], and, consequently could experience more distress during childbirth.

The World Health Organization clearly states that every woman has a right to the best attainable maternity care which should include dignity and respect, [6] and recommends emotional support during childbirth to understand her specific needs and strengthen her ability to manage the birthing process [7]. Evidence exists that women experience less distress [8], have a shorter duration of labour and reduced chances of a cesarean delivery if they are provided with support during labour and childbirth [9]. Conversely, the maternity team’s lack of awareness of a woman’s psychosocial needs could induce in her feelings of helplessness and loneliness during labour and childbirth [10]. Yet while considering the social, mental and emotional needs of women during maternity care is likely to improve their birthing experience there is a dearth of capacity-building materials for providing systematic, inclusive psychosocial support. This points to the pressing need for standardised training materials to enable maternity teams to provide psychosocial support to women during the intrapartum phase.

WHO’s Mental Health Gap Action Programme (mhGAP) [11] is an evidence-based guidance which provides psychosocial support in resource-limited health care settings.. It encompasses a detailed process of assessing mental health conditions, followed by managing them via psychosocial, pharmacological and psychological means, and following up to ascertain either recovery or need for further support. Though mhGAP identifies women of child-bearing age as a group with special needs and indicates the importance of psychosocial intervention as a first line of treatment during pregnancy, it lacks specific guidance or support for pregnant women in the intrapartum phase.

Therefore, to translate WHO’s intrapartum policy recommendations into practice, we adapted mhGAP materials for the provision of inclusive psychosocial support to women during labour and childbirth to improve their birthing experience.

The adaptation process was carried out principally under the umbrella of the Human Centered Design framework (HCD) [12–15]. HCD is used increasingly in global health to solve problems and develop systems that are useful and useable by engaging users of those systems while considering their needs, challenges and preferences. We used this framework to develop relevant and feasible psychosocial support capacity-building materials for maternity teams.

**Fig 1:**
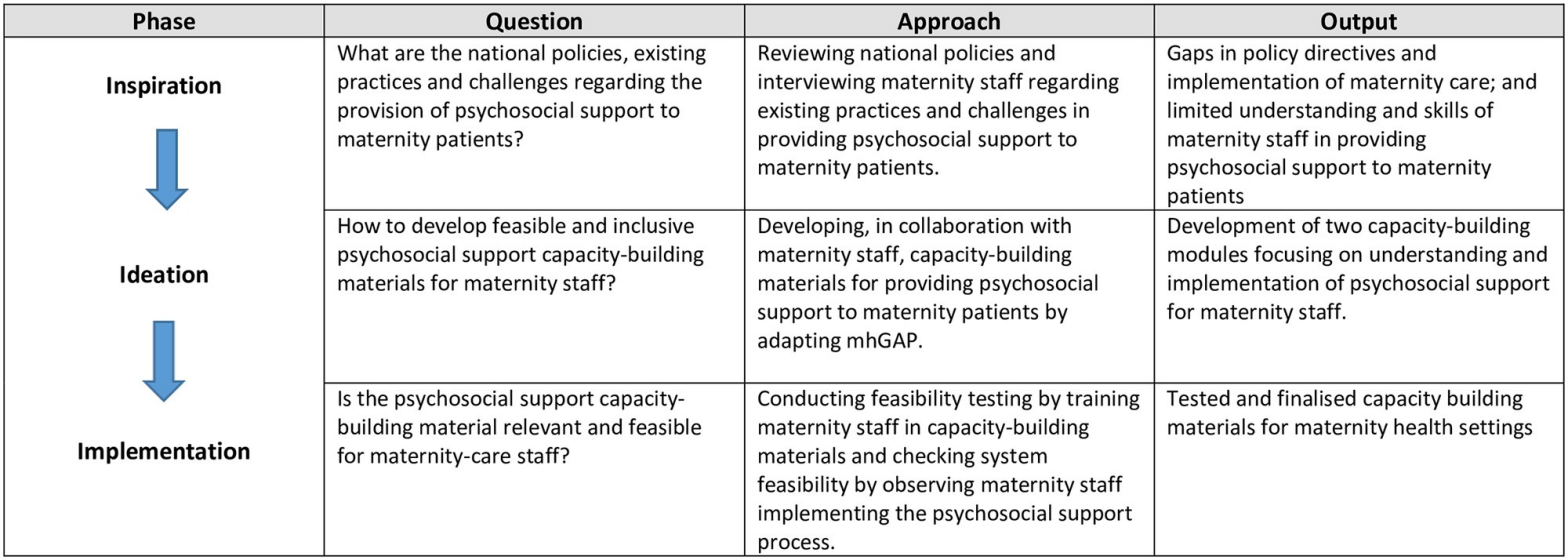
HCD’s phases with pertinent question, approach and output in the context of psychosocial support for maternity patients.

The HCD framework has three phases—*inspiration*, *ideation* and *implementation*. Each phase, in the context of supportive, inclusive and dignified care for maternity patients, had a key question with a relevant approach for adaptation and module development which led to an output (Fig 1).

This paper aims to illustrate the process of adapting mhGAP materials to develop psychosocial support capacity-building resources using the HCD framework for supporting maternity patients in the labour room. We believe this work is the first initiative to adapt mhGAP for the maternity care setting, its aim being to provide systematic psychosocial support to women to improve their birthing experience.

## Methods

### Study context

The adaptation of mhGAP to develop capacity-building materials for psychosocial support was part of a large study: Supportive and Dignified Maternity Care (SDMC). This study aimed to develop and test the feasibility of a service-delivery intervention model to promote a culture of support and respect during childbirth in public health facilities [16, 17]. Psychosocial-support training materials are part of the SDMC training handbook for maternity staff to provide supportive and dignified care to women in the intrapartum phase (during labour and childbirth—specifically from the onset of labour until completion of the third stage of labour). The capacity-building materials discussed in this paper focus specifically on the concepts, principles and implementation of inclusive psychosocial support to maternity patients, while the remainder of the materials included in the SDMC handbook have more to do with respectful treatment, elements of team building, and implementation of the SDMC strategy.

### Study setting

The adaptation process was carried out in six secondary-level public health facilities providing at least basic emergency obstetric and newborn care (BEmONC) services. These facilities were selected from the Sujawal and Thatta districts of Southern Sindh (three facilities from each district). These contiguous districts have a combined population of around 1.7 million and are approximately 95 kilometres from Karachi, the most populous metropolitan city in Pakistan. The inhabitants are predominantly Muslim (97%). Around 52% of births in Sujawal and 60% in Thatta take place in health facilities.

### Participants

Maternity staff of the labour room—clinical staff (obstetrician/gynecologist, midwife, lady health visitor, nurse, and technician) and non-clinical staff (traditional birth attendant, maid/sweeper, security guard)—participated in the adaptation process. We included non-clinical staff to consider their involvement in supporting clinical staff to provide care to maternity patients during the intrapartum phase. Health facility staff who were working in the maternity wards during the data collection period were eligible to participate in the study.

### Procedure

A multidisciplinary core team with expertise in maternal and child health, mental health and psychometric adaptation, implementation research, and social science was formed. Guided by the HCD framework, the team, planned the adaptation of mhGAP materials and the development of psychosocial support capacity-building materials in consultation with maternity staff representatives. Team ensured the systematic execution of the plan in keeping with the framework. Maternity team representatives were regularly consulted, and their input sought, through this process.

#### Adaptation of mhGAP materials and development of psychosocial support modules

**Fig 2:**
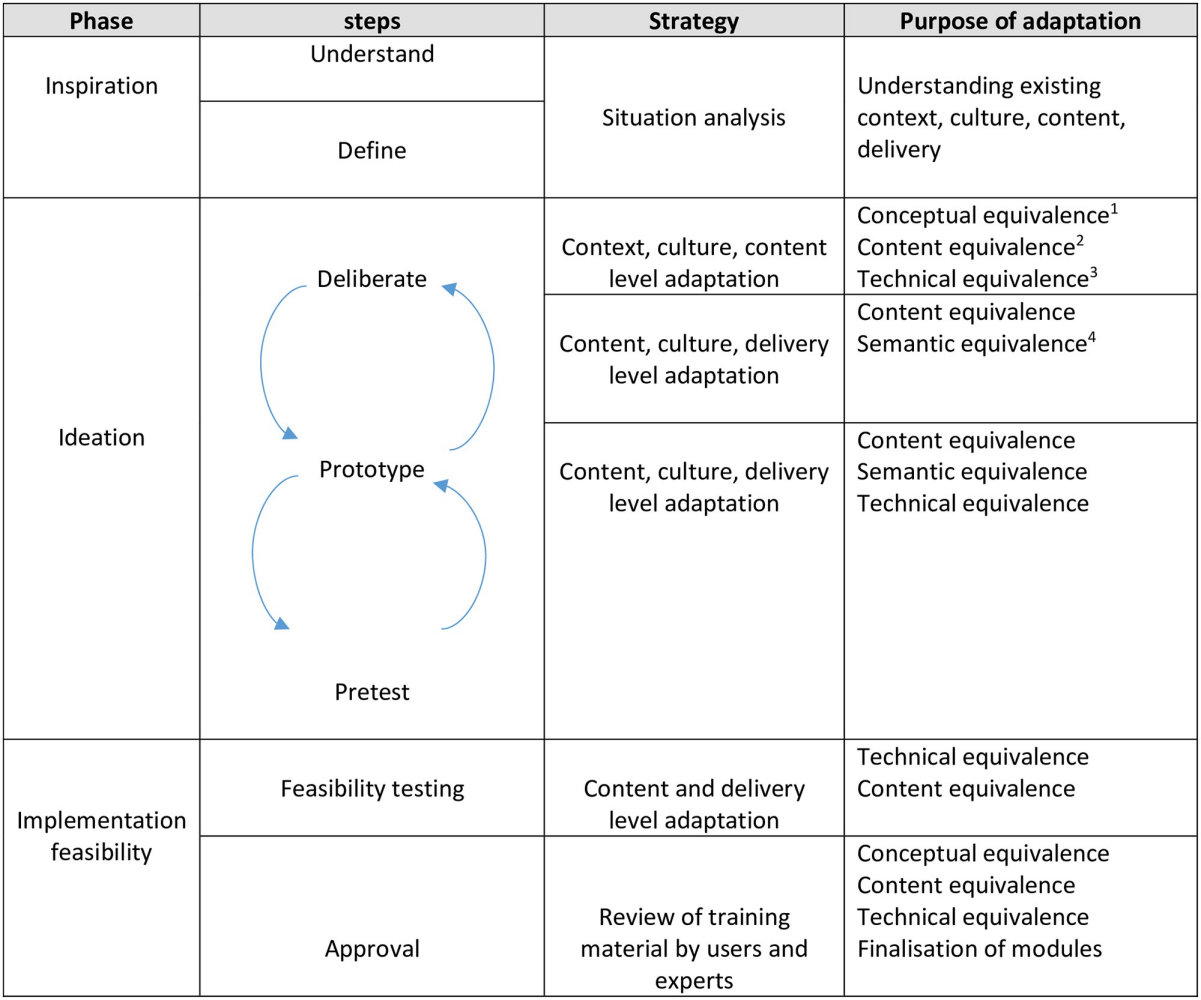
Adaptation process and its purpose. ^1^Construct equivalence refers to ensuring same concepts across cultures. ^2^Content equivalence refers to ensuring content relevance in another culture. ^3^Technical equivalence refers to ensuring same delivery of activities and implementation process cross culturally. ^4^Semantic equivalence refers to ensuring same meaning of the words cross-culturally. The Fig 2 has been adapted from the ‘Cultural adaptation model’ [18].

The adaptation of mhGAP materials to develop psychosocial support capacity-building materials was based primarily on the three systematic phases of HCD: *inspiration*, *ideation* and *implementation* [12–15], where each phase was divided into steps. In addition to this a cultural adaptation model [18]—focusing on a standardised, iterative process of adapting psychometric instruments with emphasis on maintaining methodological equivalences of content developed in another culture—was incorporated into the HCD framework to ensure equivalences of the adapted content with the original version of mhGAP and inclusion of all relevant constructs in the capacity-building materials. This was all done with mhGAP’s recommendations for adaptation in mind. Adaptation process along with the purpose of that specific process have been highlighted in the Fig 2. Below are details of each phase.

1. ***Inspiration*** focused on understanding challenges and existing practices with respect to providing psychosocial support, and on identifying relevant national guidelines. It was based on two steps, *understand* and *define*, which gave helpful structure to situation analysis.
  - **Understand**: Through in-depth interviews, a formative study was carried out (IDIs). Two trained sociologists conducted 40 interviews using an interview guide with clinical and non-clinical labour room staff as well as health managers. The existing health system’s maternity care, including psychosocial support for pregnant women, was explored through interviews [19]. The psychosocial part of the interviews explored: (i) participants’ understanding of, and current practices around, supportive care to women during labour and childbirth; (ii) the psychosocial needs of, and provision of inclusive support to, women with disabilities, mental health issues (principally anxiety and depression), or socio-demographic vulnerabilities (e.g., young age, different ethnicity, poor socio-economic status); and (iii) training that participants had received related to psychosocial support. Psychosocial support for maternity patients was defined as help—through psychological and social strategies—provided by staff to pregnant women to tackle physical, mental and emotional challenges faced during the intrapartum phase. Interviews were conducted in Sindhi language and were recorded with participants’ consent. Data were analysed in a combination of inductive and deductive approaches described elsewhere [19].
  - **Define:** Core team reviewed existing national service-delivery guidelines for maternity care during labour and childbirth to identify respectful and inclusive psychosocial support in the labour room.
    All the data and information gathered in this phase provided insights for the *ideation* phase.
2. ***Ideation*** entailed developing psychosocial support capacity-building materials in collaboration with the maternity team. It was an iterative phase composed of three steps: deliberate, prototype and pretest.
  - **Deliberate:** The core team reviewed the key findings of the *inspiration* phase with maternity team representatives for their input. This was followed by a careful review of mhGAP’s intervention guide, including its operation and training manuals, to identify relevant modules for psychosocial support in the maternity setting. WHO-recommended documents mentioned in the mhGAP guide, and those related to recommendations for the intrapartum phase, were also reviewed to identify relevant content for psychosocial-support training materials.
    Team deliberated on how best to adapt and modify the mhGAP modules while ensuring content, conceptual, semantic and technical equivalence. Inclusion of additional constructs relevant to psychosocial support in labour room settings was also considered. There were regular opportunities during this step to solicit suggestions from maternity staff representatives.
  - **Prototype (draft content and training materials)**: Based on the deliberations and suggestions for modification, a draft of capacity-building material for psychosocial support was developed.
  - **Rounds of pretesting and deliberation**: The draft capacity-building material went through two rounds of pretesting and deliberation. Pretesting entailed cognitive interviews with maternity team members, conducted by a psychologist from the core team with experience of such interviews. Pretesting sought to explore content relevance, comprehension, cultural appropriateness, and ease of delivery by maternity staff in the labour room. The first round of pretesting was done with five maternity team members from Thatta health facilities; and the second with five maternity-team members from facilities in Sujawal. Each pretesting round was followed by deliberation: the team discussed and revised the draft training materials, incorporating suggestions from the pretesting interviews. After the second round, the training materials were further revised and made ready for feasibility testing.
3. ***Implementation feasibility*** consisted of two steps: (a) feasibility testing of the materials; and (b) final approval of the materials by users and experts.
  - **Feasibility testing** was divided into (i) training of maternity staff, and (ii) exploring system feasibility.
    *Training of staff*
    We conducted five 3-day trainings of 98 labour room clinical and non-clinical staff from public health facilities in both Thatta and Sujawal districts of Sindh on all modules of Supportive and Dignified Maternity Care. Training, specifically, on psychosocial support materials lasted for one-and-a-half days, and was conducted by two core team members with a background in mental health and implementation research. Module-wise training sessions were conducted through interactive presentations and discussions, vignettes, role- plays and group activities. Training assessment was done via pre-post training quiz.
    *Exploring system feasibility*
    Post-training visits and endline qualitative interviews were conducted to assess feasibility. (i) Post-training visits to all public health facilities explored the extent to which maternity staff were able to implement the psychosocial process with ease. Through supportive supervision staff were observed across all parts of psychosocial support—including assessment, support and referral—followed by detailed discussion and feedback. (ii) 36 endline in-depth interviews of maternity staff sought to understand staff members’ overall experience of implementing psychosocial support, including challenges faced and their suggestions for improvement.
  - **Approval by users and experts**: The final draft of the psychosocial support capacity-building material—incorporating all suggestions elicited in the *implementation feasibility* phase—was shared with maternity team representatives, and with national and international experts from public and maternal health for their review.

### Ethical considerations

Participants’ written informed consent was obtained after the study’s objective, purpose and procedure had been explained to them (S1 File). The study was approved by the Institutional Review Board of the London School of Hygiene and Tropical Medicine (Reference ID: 17928) and Aga Khan University (Reference ID: 2019-1683-5607).

## Results

Consistent with the section on the study’s procedure, we have organised findings based on the HCD framework’s phases with steps, as follows:

### I. Inspiration

- **Understand**: Formative research informed us about maternity staff members’ current understanding and competencies vis-à-vis provision of supportive care, assessment of support needs, and opportunity for capacity building. What follows are significant findings with respect to psychosocial support.
  **Understanding and existing practices regarding psychosocial support**:
  ***More focus on physical health and instrumental support***: We found that maternity staff prioritised clinical care, and thus the pregnant woman’s physical health. Regarding psychosocial support, staff reported providing instrumental (tangible) support such as medicine, a wheelchair, or even giving money if the pregnant woman could not afford a fee: ‘*We give them support to walk*, *sometimes we help them when they can’t afford something*, *so we buy them whatever they need with our money*.*’* (Nurse)
  When asked specifically about emotional and social support, staff spoke of encouraging and providing support to women who cry out in fear or pain, or to those facing trauma, such as the loss of a child: *‘If a woman is scared*, *we speak to her kindly and explain the process to her*…*’* (Head nurse)
***Engagement of companion in care***
  A female companion was permitted to accompany the pregnant woman in the health facility, but was neither encouraged nor guided by maternity staff to provide any support to her. The companion’s role was usually restricted to communicating the patient’s requests to staff members and bringing her medicine or food. They also acted as an interpreter when there was a language barrier: *‘When we have a Pushto-speaking woman*, *we engage her companion to translate whatever we say to the woman*.’ (Midwife)
***Current practices and skills to determine psychosocial needs and provision of support for women***
  No specific mechanism was used to determine psychosocial needs arising from, for example, mental health issues, disability or socio-demographic vulnerability. Rather, maternity staff relied on their past experiences—and on obvious signs, such as crying—to understand when a woman in their care was suffering from anxiety. With regard to being conscious of a woman’s disability, staff only detected disability that was visible—such as the effects of polio—or that impeded communication: *‘If we notice while taking her history that a woman is deaf and dumb*, *then we ask her companion to communicate with her in sign language…’ (Head nurse)*
***Opportunity to build capacity for psychosocial support***
  Maternity staff never had any training related to psychosocial support. However, they expressed a keenness to learn how to quickly determine whether a woman was psychosocially vulnerable so that they might give her more attention and support, and refer her for appropriate treatment. But they also spoke of their lack of skills in providing psychosocial support to women in labour and childbirth, and shared their wish to learn about means of support: *‘We never had any training in providing supportive care to pregnant women … we only learned family-planning counselling during our LHV course*…*’* (Lady health visitor)
***Experience of stress and burnout in maternity staff***
  This was an unexpected theme of the interviews with maternity staff members, reported by most participants: ‘*Due to workload we do get immense stress … I get so exhausted*. *I have applied for four or five days’ leave but heads don’t approve it … eventually it affects our health … I come on duty even if I don’t feel well*…’ (Midwife)
***Need of psychosocial support for staff at the health facility***
  When pressed about the provision of psychosocial support for staff, most interviewees said there was none so they attempted to manage their stress by themselves: *‘I try to do exercise*, *it keeps my mind active and prevents burnout*…*’* (Doctor)
- **Define**: The review of national and provincial documents revealed that although there were policy directives related to patients’ rights, and to emotional and social support, system-level implementation of these policies was missing. Furthermore, the only vulnerabilities included in these policy directives were those related to age and to abuse victims; there was no mention of patient needs due to mental health, disability, or socio-demographic vulnerability [20, 21].

### 2. *Ideation*

**Deliberate**: Considering the findings of the *inspiration* phase, core team resolved to provide psychosocial support for maternity care staff too. Regarding capacity-building materials for psychosocial support, we decided to develop two modules: one that focused on understanding concepts; the other on implementation and building the capacity of maternity staff. Three mhGAP modules were identified: (i) Essential care practice (ii) Depression (iii) Other significant mental health complaints. These modules—taken from two key documents: mhGAP-intervention guide (comprising principles and clinical guidelines), and mhGAP-training of trainer manual (to train personnel on principles, clinical assessment and management)—were to be adapted for the provision of support in the labour room context. Additional mhGAP documents, recommended in the ‘Depression’ module, were reviewed to identify relevant interventions [22–24]. The rationale for module selection was as follows: ‘Essential care practice’ is a generic module dealing with principles of care and offering a structure of care provision which could be adapted for a maternity care setting. Depression, anxiety and stress—common in birthing women at maternity facilities—are the focus of the ‘Depression’ and ‘Other significant mental health complaints’ modules: we wanted to explore the relevance for our purposes of interventions suggested in both. Team deliberated on adapting and modifying the mhGAP modules in terms of content, (identifying what was relevant, or modifying as appropriate), context (particular populations, labour room setting), culture (rendering content culturally-appropriate), and delivery (how to implement content) [25]. Adhering to the cultural adaptation model throughout the adaptation process, team ensured methodological equivalence between the adapted version and the original version [18] in terms of constructs (concepts remained consistent); content (content was unaltered unless context-related modifications were needed); technical (delivery of training activities and the implementation process were the same, unless contextual modification was required); and semantics (equivalence of meaning of words across the two versions).
Below are examples related to adaptation.
***Content-related adaptation (conceptual and content equivalence)***
All psychosocial-support strategies given in mhGAP-intervention guide were included: e.g., ‘psycho-education’ (providing information about the issue); ‘reduce stress’ (guiding with strategies to lessen stress), ‘strengthen social support’ (encouraging interaction with people), ‘promote functioning in daily activities’ (encouraging them to continue regular activities) and ‘treatment planning’ (though it is not the specific intervention but it helps in discussion with patients about goals and preferences for care—an important part of the care process). Strategies were adapted with respect to patients and staff in the labour room setting.. ‘Reduce stress’ and ‘strengthen social support’ are given together in mhGAP, but we separated them to appropriate specific strategies from ‘reduce stress’. In ‘strengthening social support’, the role of the companion for the patients, and the role of colleagues and social networks for staff, was highlighted.
In a similar manner, we adapted the general principles of care from mhGAP’s ‘Essential care practices’ module by adding mention of pregnant women and/or maternity care staff, or adding relevant examples, etc. These principles were originally offered as tips: we gave them as headings in a grid, with relevant points added number-wise—for the sake of clarity and to help maternity staff remember each principle. The purpose of each principle was also included.
To build understanding and the significance of psychosocial support in the labour room context, the concepts of needs, stress, and coping were added.
Additionally, the same mhGAP structure and principles that we utilised for maternity patients were used to develop psychosocial support for staff. However, in the case of staff, the focus was particularly on burnout, i.e., a state of emotional exhaustion, cynicism and reduced professional efficacy caused by chronic workplace stress [26].
Aspects of environmental psychosocial support were also added, including maintaining cleanliness, orderliness, privacy etc. in the labour room and maternity ward.
***Context-related adaptation (semantic and technical equivalence)***
For the labour room context, the term *support* was used instead of *manage*. Our formative study found that maternity staff were willing to provide *support* during labour and delivery, including considering women’s mental health needs. *Managing* mental health conditions was beyond their expertise. Maternity staff preferred to refer women screened as depressed or anxious to a mental health specialist.
Care pathways were identified, such as: (i) a designated maternity-team member would attend to a woman’s needs if there was no companion to do so; (ii) maternity staff would consult trained senior clinical personnel within the labour room if they experienced difficulty in providing psychosocial support to the women; and (iii) a woman screened as depressed or anxious on the basis of assessment, would be referred—after delivery, at the point of exit—to the nearest mental health facility for detailed assessment and care.
***Culture-related adaptation (content and technical equivalence)***
Culturally-apt case studies and examples were added: e.g., the gender of the woman’s companion was stipulated/highlighted as female. It was noted—and endorsed by maternity staff during pretesting—that only female companions accompany women during labour and childbirth because labour room in a public sector maternity unit has 3 to 4 labour beds to conduct simultaneous deliveries and men companions are not allowed to be in the labour room due to privacy of other patients, therefore health staff suggested during pretesting that only ‘ a female’ should be mentioned as companion in the case studies to make the cases more culturally relevant.
***Delivery-related adaptation (technical equivalence)***
With respect to implementing and delivering psychosocial support at health facility level, We, as member of the Core team along with maternity-staff representatives, deliberated on a trained senior member of clinical staff at the facility overseeing SDMC-implementation, including the complete psychosocial process (assessment, support and referral). This person would be the contact person for SDMC-related activities, and be guided by the core team on implementation. They would be the contact person for guidance or support regarding psychosocial support for patients and maternity staff, and would be titled ‘mental health first aider’ (MHFA). They would also be the contact support person in the event of any trauma in the labour room setting. Importantly, the maternity team would identify who should have this role.
We also adapted training activities to make them consistent with the modules’ content, and developed training presentation slides. Training activities were added at the end of each module, while training presentation slides were for maternity-team training and would be available for refreshers. We took care that the training activities and presentation were not overly technical so that they would be accessible to all members of the maternity team, both clinical and non-clinical.
**a. Prototype (draft content and training materials)**
Psychosocial support capacity-building materials were developed into two modules, along with training activities and presentation slides. The first module, ‘Psychosocial support’, was about understanding the concepts, while the second, ‘Implementation of psychosocial support’, focused on the process of implementing psychosocial support in the health facility for pregnant women and staff separately, based on mhGAP’s structure: assessment, support and referral(see S2 File for outline of training modules).
**c. Rounds of pretesting and deliberations**
**Pretest (1st round)**
While finding all capacity-building materials primarily useful, maternity staff suggested modifications to make them more relevant to, and feasible in, the labour room context. Below are several examples.

- ***Suggestions related to module one on psychosocial support***
***Culture-related adaptation (content equivalence)***
  To support maternity staff, it was suggested that examples be added to each step of the problem-solving strategy to make the strategy more relevant.
***Content-related adaptation (content and technical equivalence)***
  Strengthening social support was one of the psychosocial strategies where we highlighted the possible role of the companion to provide care to the pregnant woman based on WHO’s recommendations [27] and suggestions given by maternity staff. Participants appreciated the idea: they had to manage work pressure and simultaneous deliveries with minimum human resources, and engaging companions in care could lessen their burden while building more rapport between woman and companion. They suggested several more activities for the companion: e.g., distracting or talking to the woman about other things to help manage her pain; acting as a translator, where called for, between the woman and maternity staff.
- ***Suggestions related to module two on implementation of psychosocial support and training materials***
***Content/context-related adaptation (content and technical equivalence)***
  Assessment of mental health is the first step of the systematic process of mhGAP intervention. Participants realised the importance of this step in determining the needs of the woman and staff, but were unwilling to follow the detailed assessment process suggested by mhGAP-intervention guide because the overwhelming burden of their current work left them no time for it, and because they considered it beyond their expertise. They suggested that we identify a quick way of determining mental health status.
***Content-related adaptation (content equivalence)***
  The entire psychosocial process (assessment, support and referral) was separate for pregnant women and staff. Participants suggested adding the purpose of each segment of the process to enhance understanding and meaningfulness: e.g., adding that the purpose of *assessment* is to identify specific needs (such as depression) so that support can be provided.
***Delivery-related adaptation (technical equivalence)***
  The relaxation technique via breathing exercise mentioned in the mhGAP was found to be very relevant and helpful by participants; however, they suggested that it be modified by including orientation to the breathing technique at the start of the exercise with a brief introduction of the steps that would be followed for better understanding and practise. They made this suggestion because the sequence of steps was not clearly mentioned in the original breathing exercise.
***Culture-related adaptation (content and technical equivalence)***
  Case scenarios were highly appreciated. Participants shared several more cases that they had experienced or observed and suggested we include them.
  ‘Strengthening social support’ is one of the psychosocial support strategy in mhGAP where we highlighted the role of companion in providing support to the pregnant woman. Participants suggested to make poster, pictorially as well as in words, about the companion’s role. They also suggested to develop a poster about the entire psychosocial support process for the patients and staff as a reminder for them to follow the steps systematically.
- **Deliberation and last round of pretesting**
  All modifications put forward by participants were discussed and incorporated.
  For psychosocial assessment for mental health issues, core team identified two globally-recognised, brief instruments for depression and anxiety [28, 29]. To ensure an inclusive psychosocial support process, a recognised yet brief functional disability instrument [30] was included, while for socio-demographic vulnerabilities we added a set of structured questions to elicit women’s status with regard to age, language, and socioeconomic circumstances. These tools were likely to meet our participants’ request for quicker psychosocial screening. Posters illustrating relevant messages were also developed.
  The revised draft materials, along with the posters, were pretested in the second round and found to be comprehensible, relevant and feasible (see S1 Fig for poster on companion support).. Participants suggested developing posters in Sindhi (the local language) too, thus ensuring better comprehension. With these suggestions incorporated, the revised version of capacity-building materials was ready for feasibility testing.

### 3. Implementation feasibility

- **Feasibility testing**
***Training of maternity staff***
  Participants found the capacity-building materials helpful and relevant. Training evaluation showed an improvement in knowledge (p-value 0.002) regarding psychosocial support. To evaluate attitudes and skills, role-plays and activities based on the implementation of psychosocial support were used and feedback for improvement was given. Participants proposed more roles for a companion to support the pregnant woman, and we later included these proposals in the modules.
  The concept of ‘mental health first-aider’ (MHFA) was very well received. Developing this concept, participants felt that, though the first-aider would be a clinical person, a non-clinical person, could also aid MHFA if needed, for example supporting woman in the absence of a companion.
  In the initial training sessions, it was challenging to cover all group-based activities in the given time. We therefore converted several activities—e.g., on the topic of anger—into discussion-based work. In doing so, we took pains to ensure that content, context and engagement of participants in the activity should not be compromised. It was reiterated across the training sessions that neither pregnant women nor maternity staff would be labeled with any mental illness as this would constitute a huge stigma in the country.
***Exploring system feasibility***
  During post-training supportive supervision visits to health facilities, maternity staff were observed and feedback was provided on strengths and areas for improvement in their implementation of psychosocial support. Our observation was that maternity staff were able, on the whole, to implement the psychosocial support process with ease. However, there was a need for more guidance on implementing psycho-education as a support strategy. Staff were guided to share key information briefly with patients or colleagues. This further validated the adaptation process.
  Endline in-depth interviews revealed that the implementation of psychosocial support in the maternity-care process enhanced staff knowledge, attitude and behaviour: in the words of a senior nurse, ‘*Now we allow one companion with the patient*. *We learned in the training that patients need psychological support and if their companions are with them they feel comforted and their fear ends … and it is also beneficial for us*, *as companions support their patients as per our suggestions*.’ (Nurse). On the subject of screening, another maternity staff said, ‘*Screening patients for mental health and disability has been very helpful*, *like when I screened a patient with anxiety I gave her more attention and support and I noticed that the patient was the calm and relaxed*.’ (Doctor)
  It was found that some staff forgot to give mental health- and well-being-related messages to women at the time of exit after delivery. Therefore, it was proposed that such messages be incorporated into the discharge card or medicine prescription sheet. This would guarantee the message was communicated, and keep the message with the woman to serve as a reminder.
  Regarding the implementation of psychosocial support for staff, no one reported any instance of burnout during the implementation phase. However, the strategy of ‘strengthening social support’ was primarily implemented, as a member of non-clinical staff attested: ‘…*I feel after the training we became more considerate about each other’s needs*, *for example if one member of staff is going to eat food then we say you can go*, *I am managing your work here … we give each other chance to relax … this is how we work now…*’ (Maid).
- **Approval by users and experts:**
  Users and experts reviewed the materials in terms of concept, content, culture, and delivery-related equivalences and appropriateness. With some minor tweaking, they deemed the materials satisfactory for use with maternity staff.

## Discussion

Respectful and quality maternity care is the right of every woman, especially during labour and childbirth, [6] and the provision of psychosocial support is an integral part of such maternity care. This paper aims to detail and elucidate the process of adapting mhGAP and developing capacity-building materials for maternity staff to practise psychosocial support in the labour room context. The process was based on the HCD framework with three key phases: *inspiration*, *ideation and implementation*. This discussion section highlights, in particular, the challenges and achievements of each phase.

The *inspiration* phase identified gaps and needs regarding psychosocial support. Our review of service-delivery documents revealed the need for systematic implementation of a respectful and inclusive psychosocial process for maternity patients in the labour room setting. Interviews with maternity staff exposed their limited knowledge and skills in providing support to pregnant women according to their physical, mental, emotional and social needs, thereby improving their birthing experience. However, their keenness to learn and to support patients paved the way for a collaborative development of capacity-building materials.

*Ideation* focused on collaboratively developing feasible psychosocial support capacity-building materials for maternity staff. mhGAP-intervention guide and training modules are meant for non-specialised personnel and could have relevance for maternity staff as well. mhGAP has been used globally, and adapted to suit various contexts, purposes, procedures, and outcomes, [31] yet there is no evidence of their having been adapted for either women in the intrapartum phase or for maternity staff. To the best of our knowledge, ours is the first effort to do this. We followed a standard approach of adaptation for evidence-based interventions and focused on adapting mhGAP materials with regard to context, content, culture and delivery [25] while considering adaptation recommendations by mhGAP [32]. Although studies do report several ways of conducting cultural adaptation of evidence based interventions [25, 33] but none focused on ensuring methodological equivalences while conducting adaptation cross-culturally. One possibility for not giving attention to it would be considering methodological equivalences limited to adaptation of psychometric instruments only. Ensuring equivalences maximizes the attainment of construct, content, semantic and operational equivalence between the original and adapted version of the materials and help in avoiding partiality and methodological biases, consequently could ensure cross-cultural validity of adapted materials. We used cultural adaptation model [18] to ensure conceptual, content, semantic and technical equivalence between the original and adapted versions which added value and scientific credibility to the adaptation process while ensuring cross-cultural consistency of the materials.

Psychosocial capacity-building materials were primarily structured into two modules. The first, ‘psychosocial support’, focused on understanding concepts, and the second, ‘implementation of psychosocial support’, dealt with the application of the support process in health facilities. The development of the materials was predicated on several key considerations. (a) We adapted selected modules from mhGAP-internvention guide and training manual because there is evidence that women in the prenatal phase are disproportionately vulnerable to experiencing depression and anxiety, [34] which may exacerbate the distress of childbirth [35]. (b) Instead of managing/treating mental health conditions, which mhGAP primarily does, our focus was on providing psychosocial support based on patients’ mental health, disability, and socio-demographic needs to improve women’s birthing experience. (c) To avoid repetition, mhGAP’s ‘Essential care and practice’ module was adapted while relevant interventions from the ‘Depression’ and ‘Other significant mental health complaints’ modules were adapted and included. (d) We didn’t include pharmacological interventions as these were not relevant for this context. (e) Previous research studies have shown how positive the role of the companion is during labour and childbirth [36]. Therefore, the companion’s role (as a psychosocial support strategy for patients) was highlighted based on recommended WHO guidelines [27] and cultural practices.

To develop a stand-alone resource for providing psychosocial support to patients and staff in maternity settings, we made various additions to the content: (a) Mindful of women’s labour and childbirth, and work-related stress for staff, we built in the concepts of stress, human needs and coping. We then added psychosocial support, from mhGAP, as a systematic process to enable individuals to cope with stress. (b) There is ample evidence of maternity staff experiencing work-related stress and burnout [37]. The need for psychosocial support for them also emerged in the *inspiration* phase. We therefore added the concept of burnout. Thus we were able to highlight the needs and stress-experiences of staff, and suggest supportive means—primarily identified from mhGAP—of managing stress and averting burnout. This addition could be relevant for health staff working in settings other than the labour room. (c) Connecting theory with practice, we included clinical vignettes expressing manifestations of different mental health conditions and behaviours in patients and maternity staff. (d) Posters related to the psychosocial support process for patients and staff, and to companion engagement, were developed to serve as a cue to action. We were particularly mindful in developing illustrations for the posters, taking inspiration from the artwork of Henri Matisse [38], we ensured that the human images on our posters appeared universal, rather than recognisable on the basis of colour, physical features or signifiers of socio-economic status. (e) To make the psychosocial support modules experiential, relevant training activities from the mhGAP training manual were adapted for maternity settings.

Constructs included in the modules are universal in nature and could have equal value cross-culturally, while implementation of these concepts may vary according to different health systems. The *implementation feasibility* phase focused on building the capacity of maternity staff and determining the feasibility of the psychosocial support process in health systems. There were various challenges to overcome in this phase to ensure system feasibility. (a) Unlike the assessment given in mhGAP, internationally-recognised brief instruments were used: staff did not have the time for detailed assessments and differential diagnoses but were amenable to quick screening to identify needs. The instruments used are globally recognised and psychometrically sound instruments [28, 29], thus ensuring conceptual equivalence of the assessment process. (b) The structure of the psychosocial support process for patients and staff was the same (to avoid confusion) although details differed between the two. (c) Care pathways were identified as follow-up, but were not feasible in the current context. Referral to a specialist for further assessment and support was included when a patient was positively screened for depression or anxiety. The efforts of the maternity team to make referrals based on screening instruments could be considered an important contribution to the prevention of mental illness and promotion of mental health. (d) The labour room team was composed of clinical and non-clinical staff. Where there were too few members of clinical staff, non-clinical team members typically provided support during delivery. Given the precedent for task-sharing, non-clinical staff could support a woman who has no companion. Most importantly, they could play a vital role in ensuring environmental psychosocial support by making the physical space of the labour room and ward clean, organised and welcoming. (e) To ensure successful implementation of psychosocial support at facility level, a mental health first-aider (MHFA) was introduced. This person would be a member of clinical personnel and the principal trainer in psychosocial support. They would not only ensure the implementation of the psychosocial support process for patients and staff, but also oversee all SDMC-related implementation, and be available to provide support to patients and colleagues at moments of trauma. They could also run psychosocial support refresher-trainings for staff.

Psychosocial support materials are a capacity-building resource for health staff working in maternity settings. Based on feasibility testing, a two-day experiential training course could be designed for clinical and non-clinical staff. Since these groups of staff work together as a team, non-clinical staff may benefit from considering principles of care such as promotion of respect and dignity, and effective communication, and the provision of general support when a companion is unavailable. They would also likely benefit from training on the implementation of environmental psychosocial support in the labour room and maternity ward. Our work is an attempt to implement WHO-policy recommendations for respectful maternity care by developing guidance to provide a systematic psychosocial support system in the labour room context. In terms of future utility and generalizability of our work, these materials can be used in similar health settings after minor tweaking related to cultural adaptation. Whereas for different health systems we have laid down the methodology which might be useful for adaptation of mhGAP according to that setting.

Our research was limited by omitting psychological interventions included in mhGAP. The non-specialised personnel for whom we developed the modules didn’t have sufficient time for such complex and lengthy processes. Also, patients rarely return for postnatal health follow-up, and psychological interventions may not be very relevant in this case because they require multiple long sessions. A future possibility would be to check the feasibility of psychological interventions by building the capacity of labour-room staff to help colleagues in need. Moreover, the feasibility of psychosocial support capacity-building materials can be tested in different contexts and at different levels of the health systems.

## Conclusion

Providing supportive care to pregnant women during labour and childbirth based on their psychosocial needs could make their birthing experience a positive one. We adapted the mhGAP to develop capacity-building materials for the provision of systematic psychosocial support to women in the intrapartum phase, and also for maternity staff to prevent stress and burnout. Our materials extend the utility of mhGAP to the maternity setting and can be used for capacity building of maternity staff.

## Supporting information

S1 File Informed consent form. (PDF)

S2 File Psychosocial support capacity building materials for maternity staff: Structure, content and purpose. (PDF)

S1 Fig Poster for companion support. (TIF)
