# Supplementary material for: Psychosocial support during childbirth: Development and adaptation of WHO’s Mental Health Gap Action Programme (mhGAP) for maternity care settings
Source: PLoS One. 2023 May 22;18(5):e0285209. doi: 10.1371/journal.pone.0285209 (PMC10202270; doi:10.1371/journal.pone.0285209)
Supplement: S1 File — (PDF) [file pone.0285209.s001.pdf]

Participant Information Sheet

Title of Project: Supportive and dignified maternity care in public health facilities (SDMC)

Introduction:
Assalam u alaikum! My name is __________________, and I'm associated with the Aga Khan University. I am conducting this interview as part of the research being conducted to develop and test feasibility of an intervention package that promote supportive and dignified maternity care in public health facilities. We would like to invite you to take part in a research study. Joining the study is entirely up to you. Before you decide, you need to understand why the research is being done and what it would involve. I would like to give you an overview of this study and invite you to be part of it. I will read this form to you. Ask questions if anything you read is not clear or you would like more information. Please feel free to talk to others about the study if you wish. Take time to decide whether or not to take part.

Purpose:
The London School of Hygiene and Tropical Medicine (LSHTM) are conducting this research to develop service delivery package using participatory approach and test its feasibility in public health facilities. The purpose of this interview is to understand challenges and opportunities around provision of supportive and dignified maternity care. We will explore your perceptions of working conditions, responsibilities, motivation toward their job, empathy with and prejudice against patients, availability and awareness of institutional policy and guidelines pertaining to ensuring patient-centred care. We will also gather your opinions and suggestions regarding possible approaches/strategies that could address mistreatment during childbirth. The information you provide will help us develop the package to promote supportive and dignified maternity care at public health facilities.

You have been invited because you are part of this health facility and associated with maternity care services which is the focus of this research. We ask you and about few more staff members who work at selected public health facilities.

Voluntary Participation:
You will not receive any money or other gifts for taking part in this study; however, you may be invited to attend a training work to be organised under this research project. Your participation in this interview is completely voluntary. It is your choice whether or not to participate in the study. If you choose not to participate, that's ok. You will not be treated any differently at your work. We will discuss the study together and give you a copy of this information sheet. If you agree to take part, we will then ask you to sign a consent form.

Procedure:
After you are enrolled in this study, we can start the interview right away; the interview will take about 50-60 minutes. The interview will be audio-recorded for transcription, analysis and report writing. You do not have to do anything other than the time you spend with us. This information will not be used to identify you specifically thereby it will not be possible for anyone to know what you have said. Only the study investigators will have access to hard and soft copies of the data; however, your name/identity will be kept anonymous. Please let me know if anyone has any reservation or want me to explain anything that you do not understand.

Possible Risks:
There are no foreseeable risks involved in participating in this study, except for confidentiality loss. However, the chances are very low and we will take several measures to minimise this risk such as contact details will be stored separately, names or any personal identifier will not be reflected in any report, data files will be password protected and only authorised personnel will be access to the information we collect from you. All study staff will protect the

confidentiality of participants to the fullest extent possible, except as required by law, in order to protect the rights and welfare of the participants. Therefore, I want you to feel comfortable sharing your experiences, opinions and perceptions with me. If, at any time you do not feel comfortable answering a particular question, you can choose not to answer the question.

Benefits:

There are no direct benefits to you for participating in this discussion, but information you share will help us understand the quality of care provided at the public health facilities and we hope to use this information to improve the quality of care at public health facilities. However, as I said you may be invited to participate in a training workshop to be organised under this project on supportive and dignified care.

If You Have Questions or a Problem

If you have a concern about any aspect of this study, you should ask to speak to the researchers who will do their best to answer your questions, please call Waqas Hameed at +92-3343114410, or by email waqas.hameed@aku.edu. If you remain unhappy and wish to complain formally, you can do this by contacting Patricia Henley at rgio@lshtm.ac.uk or +44 (0) 20 7927 2626.

Right to refuse or withdraw:

Even if you choose to participate, you will have every right to refuse to answer any question - which you may not want to - during the interview or withdraw at any point from the study without any explanation or penalty. If you withdraw from the study we will destroy all your personal information, but we will need to use the data collected on you up to your withdrawal.

Compensation:

You will not be compensated for your participation in this research.

Confidentiality:

All information collected about you will be kept private. It will not be possible to identify you from any information we release or use. We will not discuss your individual answers with staff members. Only the study staff and authorities who check that the study is being carried out properly will be allowed to look at information about you. Data may be sent to other study staff in London but this will be anonymised. This means that any information about you which leaves the hospital/clinic, will have your name and address removed so that you cannot be recognised.

At the end of the project, the study data will be archived at Aga Khan University and London School of Hygiene and Tropical Medicine. The data will be made available to other researchers worldwide for research and to improve medical knowledge and patient care. Your personal information will not be included and there is no way that you can be identified.

The study results will be published in a medical journal so that other doctors can learn from them. Your personal information will not be included in the study report and there is no way that you can be identified from it.

Aga Khan University is the sponsor for the research and they have full responsibility for the project including the collection, storage and analysis of your data.

Ethics review:

All research involving human participants is looked at by an independent group of people, called a Research Ethics Committee, to protect your interests. This study has been reviewed and given favourable opinion by The London School

of Hygiene and Tropical Medicine Research Ethics Committee (URL: https://www.lshtm.ac.uk/research/research-governance-integrity/ethics). The Ethics Review Committee of the Aga Khan University has also reviewed the study and have agreed that it is okay for us to ask people to take part.

Further information and contact details:
Thank you for your attention as I read out this information leaflet. If you think you will take part in the study please read and sign the consent form. If you would like any further information, please contact Waqas Hameed on below mentioned contact details who can answer any questions you may have about the study.

Contact details:
Waqas Hameed
Phone: +92-334-3114410
Email: waqas.hameed@aku.edu

Do you have any questions at this time?

Thank you for your time.

Title of Project: Supportive and dignified maternity care in public health facilities (SDMC)
Name of PI/Researcher responsible for project: Dr Bilal Iqbal Avan

Statement	Please initial or thumbprint* each box
I confirm that I have read and understood the information sheet version 1.0 dated 29-August-2019 for the above named study. I have had the opportunity to consider the information, ask questions and have these answered satisfactorily.	
I understand that my consent is voluntary and that I am free to withdraw this consent at any time without giving any reason and without my/the participant's medical care or legal rights being affected.	
I understand that relevant sections of my/the participant's medical notes and data collected during the study may be looked at by authorised individuals from Aga Khan University and London School of Hygiene and Tropical Medicine, where it is relevant to my/the participant's taking part in this research. I give permission for these individuals to have access to these records.	
I understand that data about/from me/the participant may be shared via a public data repository or by sharing directly with other researchers, and that I will not be identifiable from this information	
(Include if applicable) I agree to my/the participant's GP being informed of my participation in the study.	
I agree to me/the participant taking part in the above named study.	

**Printed name of participant/Representative**	**Signature of participant/Representative** (or thumbprint/mark if unable to sign)	**Date**
**Printed name of person obtaining consent**	**Signature of person obtaining consent**	**Date**

The participant/representative is unable to sign. As a witness, I confirm that all the information about the trial was given and the participant/representative consented to taking part *(*only required if the participant/representative is unable to read or write)*

**Printed name of impartial witness***	**Signature of impartial witness***	**Date**

A copy of this informed consent document to be offered to the participant
