# Supplementary material for: Psychosocial support during childbirth: Development and adaptation of WHO’s Mental Health Gap Action Programme (mhGAP) for maternity care settings
Source: PLoS One. 2023 May 22;18(5):e0285209. doi: 10.1371/journal.pone.0285209 (PMC10202270; doi:10.1371/journal.pone.0285209)
Supplement: S2 File — (PDF) [file pone.0285209.s002.pdf]

Annexure

This annexure highlights the psychosocial support capacity-building materials' module-wise key contents and their purpose

Psychosocial support capacity building materials for maternity staff: Structure, content and purpose

Module	Content	Purpose
**Psychosocial Support Module 1**	1.1 Core concepts:<br>- Human needs<br>- Stress<br>- Coping<br>- Psychosocial support	To orient the participants with the key concepts related to psychosocial support. Also these concepts have been mentioned frequently in the module, therefore explaining them in the beginning of the session gives clarity to the participants and enhance their engagement in learning process.
	1.2 Team dynamics, needs, stress and coping resources in maternity care staff<br>- Needs and stress experience of staff<br>- Burnout and its consequences	Its purpose is to highlight the stress experience of maternity team. Particularly, the concept of burnout has been introduced with the focus on its precursors and also the consequences.
	1.3 Needs and stress experience of pregnant woman<br>• Experience of depression and anxiety	Its purpose is to develop an understanding of the participants that besides physical health related needs, maternity patients have mental, emotional and social needs that are important to address to ensure their positive birthing experience. This section specifically mentions the needs of patients who may have mental health issues, disability or any socio-demographic vulnerability.
	1.4 General principles of care in the context of SDMC<br>• Promote respect and dignity<br>• Use of effective communication	To develop understanding of the principles of care to provide supportive and respectful maternity care. Understanding of these principles would support in the implementation of the psychosocial support.
	1.5 Psychosocial support strategies - equally applicable to maternity staff and pregnant women<br>Psychosocial support strategies for maternity staff<br>• Psychoeducation<br>• Reduce stress (Breathing exercise & Problem solving technique)<br>• Strengthen social support (Identifying supportive people at work and home)<br>• Promote scheduling in daily professional activities<br>Psychosocial strategies for pregnant woman<br>• Psychoeducation (Labour and childbirth & Needs and assessed conditions)<br>• Reduce stress (Breathing exercise)<br>• Strengthen social support (Engaging companion in care provision)<br>• Promote functioning in daily activities	Purpose of this section is to introduce different psychosocial strategies for maternity staff and maternity patient. It emphasizes that both maternity staff and patients need support to have better well-being. The concept and the purpose of these strategies are same but these differ in terms of implementation with the maternity team and the patient.
	Activities	This module has 4 activities pertaining to principles of care and psychosocial support and are conducted using case studies. The purpose of these activities is to encourage participants understand and critically analyze cases/situations and suggest appropriate ways to respond to them.

<table>
<tr><th>Module</th><th>Content</th><th>Purpose</th></tr>
<tr><td rowspan="3">Implementation of Psychosocial Support Module 2</td><td>2.1 Core concepts
• Levels of implementation of psychosocial support
• Environmental level
• Individual level
• Trauma and experience of grief in the labour room setting</td><td>To orient the participants with the key concepts related to implementation of psychosocial support. Also these concepts have been mentioned frequently in the module, therefore explaining them in the beginning of the session gives clarity to the participants and enhance their engagement in learning process.</td></tr>
<tr><td>2.2 Specific steps in providing psychosocial support to maternity staff
• Assessment
o Physical health assessment
o Psychosocial assessment (burnout, anxiety and depression)
• Support
o Physical health support (if urgent)
o Psychosocial support
- Psychosocial care planning
- Psychoeducation
- Reduce stress
- Strengthening social support
- Promote everyday scheduling and lifestyle changes

Referral
▪ If symptoms persists</td><td>This section focuses on implementation of psychosocial support process to provide support to maternity staff. Its purpose is to build skills of the maternity team to follow a systematic process to assess needs of their colleague and provide them support to cope with stress.</td></tr>
<tr><td>2.3 Specific Steps of providing psychosocial support to pregnant women in intrapartum phase
• Assessment
o Physical health assessment
o Psychosocial assessment (anxiety, depression and disability)
• Support
o Physical health support
o Psychosocial support
- Psychosocial care planning
- Psychoeducation
- Reduce stress
- Strengthening social support
- Promote everyday scheduling and lifestyle changes

• Referral
▪ At the time of exit, if score of depression or/and anxiety is 3 or more</td><td>This section focuses implementation of psychosocial support process to provide support to maternity patient. Its purpose is to build skills of the maternity team to follow a systematic process to assess needs of the patients and provide them support during labour and childbirth so that they have positive birthing experience.</td></tr>
</table>

	2.4 Psychosocial support to deal with trauma and grief in labour room setting for pregnant women and maternity care team	The purpose of this section is to build capacity of the maternity team to provide support to patient in case of them experiencing any traumatic event pre,inter and postpartum phase to help them cope with traumatic experience. It also provide guidance about providing support to maternity team in case of experiencing any traumatic event in the labour room setting.
	Activities	This module has 3 activities focusing on practicing psychosocial support process including assessment, support and referral for patient and staff. The purpose of these activities are to build skills of the participants with respect to provision of psychosocial support process.
