# Supplementary figures and images for: Psychosocial support during childbirth: Development and adaptation of WHO’s Mental Health Gap Action Programme (mhGAP) for maternity care settings
Source: PLoS One. 2023 May 22;18(5):e0285209. doi: 10.1371/journal.pone.0285209 (PMC10202270; doi:10.1371/journal.pone.0285209)

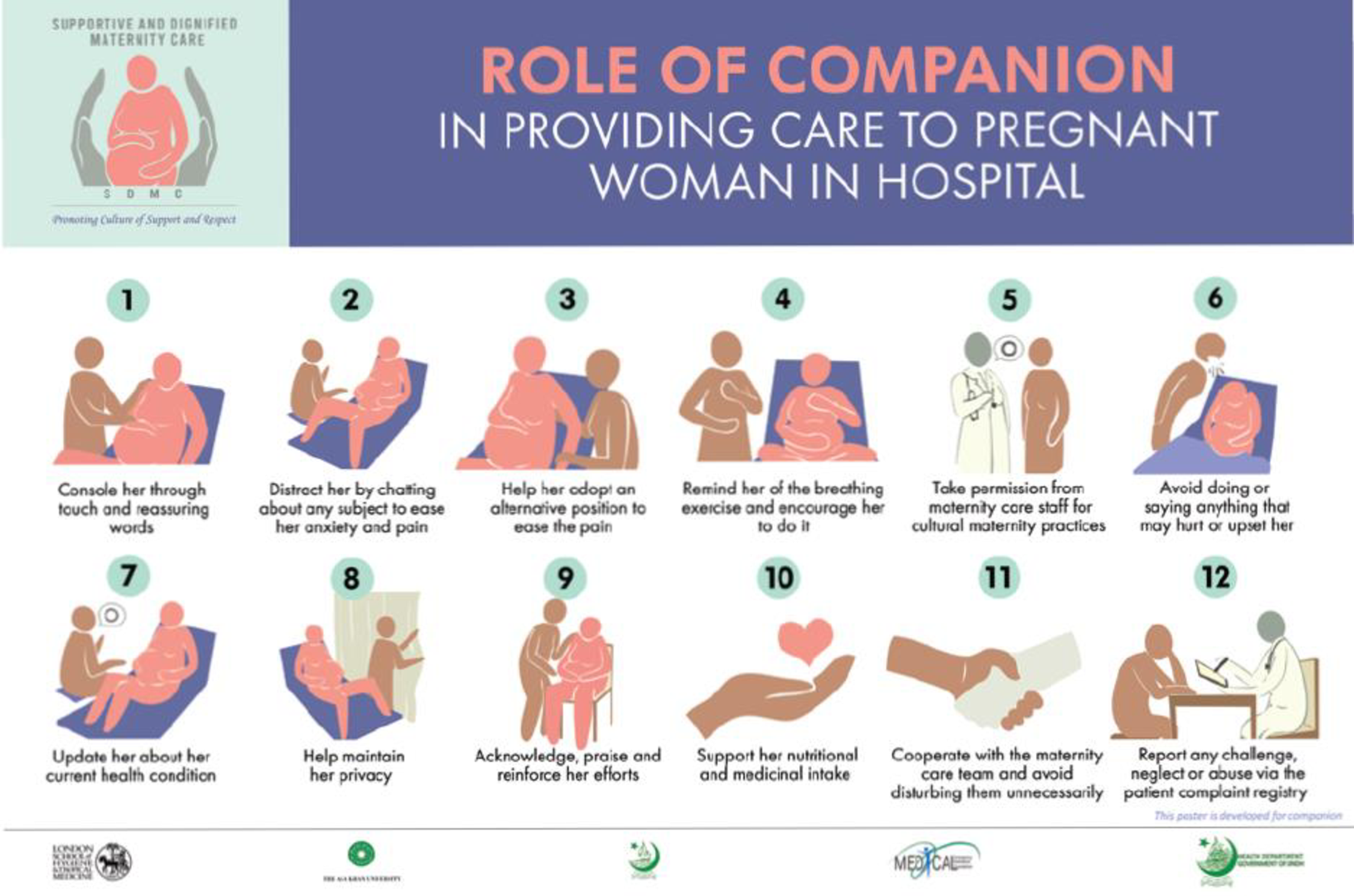

Supplement: S1 Fig — (TIF) [file pone.0285209.s003.tif]
